# Supplementary figures and images for: A Five Collagen-Related Gene Signature to Estimate the Prognosis and Immune Microenvironment in Clear Cell Renal Cell Cancer
Source: Vaccines (Basel). 2021 Dec 20;9(12):1510. doi: 10.3390/vaccines9121510 (PMC8707639; doi:10.3390/vaccines9121510)

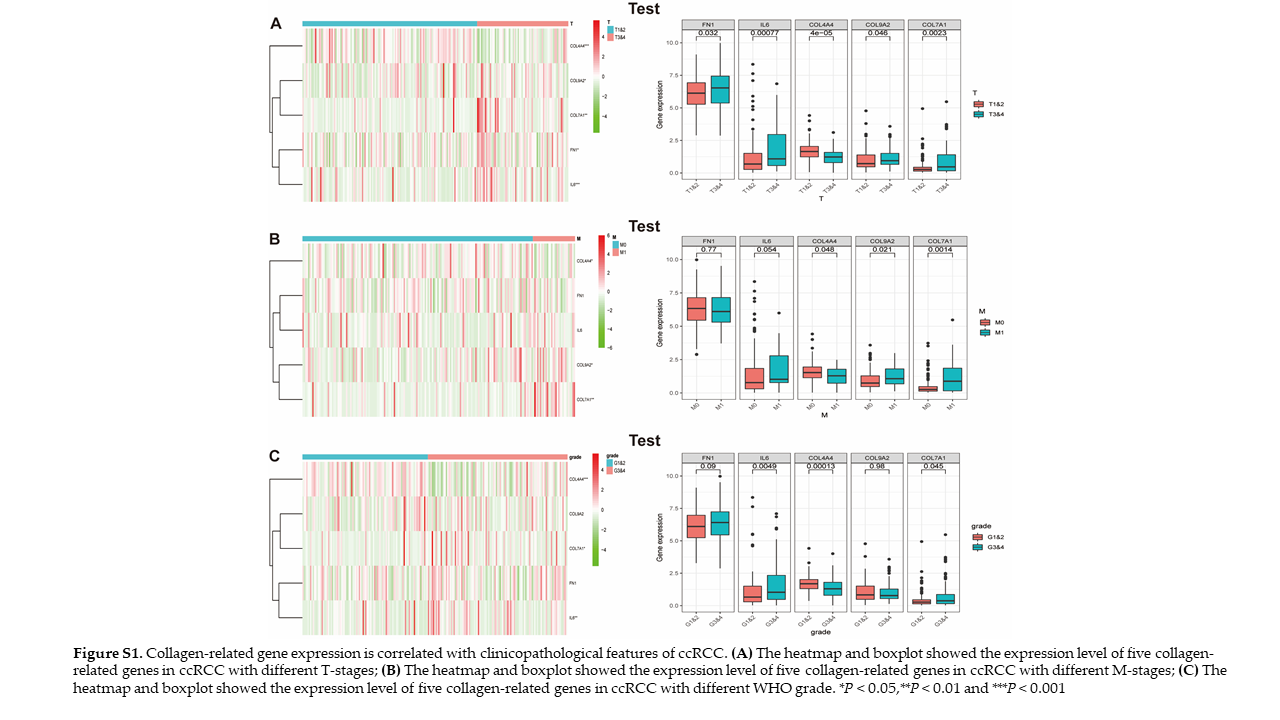

Supplement: Supplementary file 1 [file vaccines-09-01510-s001.zip › vaccines-1495533-supplement-final-updated/Figure S1.tif]
